# PReS-FINAL-2224: Canakinumab treatment regimens in CAPS-patients

**DOI:** 10.1186/1546-0096-11-S2-P214

**Published:** 2013-12-05

**Authors:** F Hofer, T Endres, B Kortus-Götze, N Blank, E Weißbarth-Riedel, C Schütz, T Kallinich, K Krause, C Rietschel, G Horneff, J Kuemmerle-Deschner

**Affiliations:** 1 Department of Pediatrics, Division of Pediatric Rheumatology, University Hospital Tuebingen, Tuebingen, Germany; 2 Klinik für Innere Medizin, Klinikum der Philipps-Universität Marburg, Marburg, Germany; 3 Hämatologie, Onkologie u. Rheumatologie, Universitätsklinikum Heidelberg, Heidelberg, Germany; 4 Kinderrheumatologische Ambulanz, Universitätsklinikum Eppendorf, Hamburg, Germany; 5 Klinik für Kinder und Jugendmedizin, Universitätsklinikum Ulm, Ulm, Germany; 6 Kinderklinik Sektion Rheumatologie, Charité Campus Virchow, Berlin, Germany; 7 „Allergie-Centrum Charité", Klinik für Dermatologie, Charité Campus Mitte, Berlin, Germany; 8 Kinder-und Jugendrheumatologie, Clementine-Kinderhospital, Frankfurt, Germany; 9 Abteilung für Allgemeine Kinder-und Jugendmedizin, Asklepios-Klinik Sankt Augustin, Sankt Augustin, Germany

## Introduction

Canakinumab is a recombinant monoclonal fully human antibody against Interleukin-1β and approved for the treatment of CAPS in many countries including Europe and the US. Current dose recommendations are 150 mg (body weight >40 kg) respectively 2 mg/kg body weight (15 to 40 kg) every 8 weeks but yield insufficient response in some individuals, especially in children and patients with severe phenotypes [1].

## Objectives

In this study we analyzed the response to daily practice (in contrast to trial condition) canakinumab treatment regimens in CAPS patients with focus on age, mutation and clinical presentation and the necessity and effect of dose adjustment.

## Methods

An observational national multicenter study was conducted. CAPS patients were included if they received at least two doses of canakinumab. Data included information regarding demographics, treatment, clinical disease activity and inflammatory markers (including SAA, CRP, ESR, IL-6). Response to treatment was assessed using CAPS-disease activity scores, CRP and/or SAA levels.

## Results

A cohort of 68 patients with CAPS was analyzed. Median age was 25.4 years (range 22 months to 73 years). When treatment was initiated, 27 patients had been younger than 18 years. The most frequent mutations were R260W, A439V, E311K, V198M, Q703K and most patients showed MWS or FCAS/MWS phenotype (3 patients with NOMID, 4 with MWS/NOMID). The median treatment duration was 855 days (range: 28-1973 days). In 39 patients (57%) full response was sustained until the next scheduled drug application (34% (23 patients) partial remission). With standard treatment 21 patients (31%) achieved full response. In 30 patients (44%) canakinumab dose and/or application interval was increased above the standard regimen (2/3 NOMID, 3/4 MWS/NOMID). Neither laboratory parameters nor clinical disease activity at the beginning of treatment were able to predict the necessity to adjust treatment regimen. Two serious adverse events were reported (severe infection, osteonecrosis), mild and moderate adverse events were mostly upper respiratory tract infections but almost no injection site reactions.

## Conclusion

Most CAPS patients achieve full remission with canakinumab. However, almost 50% of patients, particularly children, require dose adjustment. Full remission by dose increase was achieved without an increased rate of adverse events. Individual adjustment of therapy should be performed as needed as predictive parameters are lacking.

## Disclosure of interest

F. Hofer: None declared, T. Endres: None declared, B. Kortus-Götze: None declared, N. Blank: None declared, E. Weißbarth-Riedel: None declared, C. Schütz: None declared, T. Kallinich: None declared, K. Krause: None declared, C. Rietschel: None declared, G. Horneff: None declared, J. Kuemmerle-Deschner Grant/Research Support from: NOVARTIS, Consultant for: NOVARTIS, SOBI.
